# Harmonization of the Volume of Interest Delineation among All Eleven Radiotherapy Centers in the North of France

**DOI:** 10.1371/journal.pone.0150917

**Published:** 2016-03-17

**Authors:** David Pasquier, Laurence Boutaud de la Combe-Chossiere, Damien Carlier, Franck Darloy, Anne Catherine Degrendel-Courtecuisse, Chantal Dufour, Mustapha Fares, Laurent Gilbeau, Xavier Liem, Philippe Martin, Pascal Meyer, Jean François Minne, Olimpia Olszyk, Hassan Rhliouch, Marc Tokarski, Chloé Viot, Bernard Castelain, Eric Lartigau

**Affiliations:** 1 Academic Radiation Oncology Department, Centre Oscar Lambret, Lille University, Lille, France; 2 CRISTAL, UMR CNRS 9189, Lille, France; 3 Institut Andrée Dutreix, Dunkerque, France; 4 Centre Léonard de Vinci, Dechy, France; 5 Centre Joliot Curie, Boulogne sur mer, France; 6 Centre de Cancérologie Les Dentellières, Valenciennes, France; 7 Centre Pierre Curie, Béthune, France; 8 Centre Gray, Maubeuge, France; 9 Centre Bourgogne Clinique du Bois, Lille, France; 10 Centre Galilée, Lille, France; 11 Centre Marie Curie, Arras, France; 12 Centre de Cancérologie de l’Artois, Lens, France; 13 Réseau Onco Nord Pas de Calais, Loos, France; Hunter College of The City University of New York, UNITED STATES

## Abstract

**Background:**

Inter-observer delineation variation has been detailed for many years in almost every tumor location. Inadequate delineation can impair the chance of cure and/or increase toxicity. The aim of our original work was to prospectively improve the homogeneity of delineation among all of the senior radiation oncologists in the Nord-Pas de Calais region, irrespective of the conditions of practice.

**Methods:**

All 11 centers were involved. The first studied cancer was prostate cancer. Three clinical cases were studied: a low-risk prostate cancer case (case 1), a high-risk prostate cancer case (pelvic nodes, case 2) and a case of post-operative biochemical elevated PSA (case 3). All of the involved physicians delineated characteristically the clinical target volume (CTV) and organs at risk. The volumes were compared using validated indexes: the volume ratio (VR), common and additional volumes (CV and AV), volume overlap (VO) and Dice similarity coefficient (DSC). A second delineation of the same three cases was performed after discussion of the slice results and the choice of shared guidelines to evaluate homogenization. A comparative analysis of the indexes before and after discussion was conducted using the Wilcoxon test for paired samples. A p-value less than 0.05 was considered to indicate statistical significance.

**Results:**

The indexes were not improved in case 1, for which the inter-observer agreement was considered good after the first comparison (DSC = 0.83±0.06). In case 2, the second comparison showed homogenization of the CTV delineation with a significant improvement in CV (81.4±11.7 vs. 88.6±10.26, respectively, p = 0.048), VO (0.41±0.09 vs. 0.47±0.07, respectively; p = 0.009) and DSC (0.58±0.09 vs. 0.63±0.07, respectively; p = 0.0098). In case 3, VR and AV were significantly improved: VR: 1.71(±0.6) vs. 1.34(±0.46), respectively, p = 0.0034; AV: 46.58(±14.50) vs. 38.08(±15.10), respectively, p = 0.0024. DSC was not improved, but it was already superior to 0.6 in the first comparison.

**Conclusion:**

Our prospective work showed that a collaborative discussion about clinical cases and the choice of shared guidelines within an established framework improved the homogeneity of CTV delineation among the senior radiation oncologists in our region.

## Introduction

The Nord-Pas de Calais region is the fourth most populated region of the 22 French metropolitan regions, with 4.052 million inhabitants. This region includes the North and Pas-de-Calais departments and represents 6.2% of the French population. It is one of the most densely populated regions, with 326 inhabitants/km^2^ compared with 115 inhabitants/km^2^ in metropolitan France. In Lille and the surrounding areas, where the seat of the Regional Council of Nord-Pas-de-Calais is located, a comparison with national data shows an increased incidence of head and neck, esophageal, lung, liver, bladder, kidney, colorectal, uterine and ovarian cancers [1]. This region comprises 11 centers of radiotherapy.

Radiotherapy plays a key-role in the treatment of cancer. The highly conformal dose distributions produced using modern techniques require careful delineation of target volumes and organs at risk (OARs). An inadequate radiotherapy plan can diminish the chance of a cure and/or increase the risk of toxicity. The quality of radiotherapy plans affects the outcome of chemo radiotherapy in head and neck cancer [2]. In a meta-analysis of eight studies (4 pediatric and 4 adult patients) the frequency of quality assurance deviations ranged from 8% to 71% and radiotherapy deviations were associated with a statistically significant decrease in overall survival (HR of death = 1.74, 95% confidence interval [CI] = 1.28 to 2.35; p < .001) [3].

Prostate cancer is the second most common cancer in men and remains the most common cancer in developed countries [4]. Inter-observer variability in the definition of target volumes has been well established since the beginning of conformal and intensity-modulated radiotherapy for prostate cancer [5–7]. The aim of our work was to improve the delineation homogeneity among the radiation oncologists in the Nord-Pas de Calais region through collaborative discussions concerning clinical cases and the selection of shared guidelines.

## Materials and Methods

All 11 centers were involved: eight private, two with mixed public-private activity and one academic department of radiation oncology. The first studied cancer was prostate cancer. Three fictitious clinical cases were sent to all of the centers. Each case included a detailed description of the clinical history, histologic or anatomopathologic data and computed tomodensitometry (CT) scan of anonymized images. Low- and high-risk (pelvic nodes) prostate cancer according to the D’Amico classification and post-operative biochemical elevated PSA cases were studied. In case 1, anonymized magnetic resonance (MR) images for image fusion were also sent. A detailed description of the three cases and the volumes to be delineated is presented in Table 1. These data were sent with the P2E (AQUILAB SAS) workstation that equips each center. All of the involved physicians delineated characteristically the clinical target volume (CTV) and OARs. After delineation, each center sent the data to Onco-npdc, where contours were compared (C. Viot); the delineation was also anonymized. The following validated indexes were used for delineation comparison: the volume ratio (VR), common and additional volumes (CV and AV), volume overlap (VO) and Dice similarity coefficient (DSC) (Table 2) [8–12]. The contours of a participant were randomly selected as the “reference” (method 1). The same participant was selected for all three cases during the two comparisons. Indeed, the aim of the present study was to increase the homogeneity of delineation, and we hypothesized that the choice of the “reference” contours did not significantly influence the results. We compared each contour with a common contour also comprising the delineation of 9/14 physicians (method 2). This method facilitates the evaluation of delineation harmonization and avoids the selection of a random or reference contour [13]. The results were discussed slice by slice by senior and junior radiation oncologists during three meetings a year, and shared guidelines were selected for each clinical case. A second delineation of the same three cases was then performed to quantify the standardization. The first delineation was conducted during the month prior to the meeting, and the second delineation was achieved in the month following the meeting. The same methodology and indexes of the first comparison were used. Comparison of the OAR delineation was not realized. Statistical analyses were performed using JMP® (Version 10; SAS Institute Inc., SAS Campus Drive, Cary, North Carolina). A comparative analysis of the index before and after discussion was performed using the Wilcoxon test for paired samples. The VO and DSC of the three cases were also compared (Mann-Whitney test for unpaired samples). A p-value less than 0.05 was considered to indicate statistical significance.

**Table 1:** Description of the three clinical cases.

	Clinical case	Sent images	Volumes to be delineated
**Case one**	**Low-risk prostate cancer**. PSA = 8 ng/ml, T1cN0 stage. Histology: adenocarcinoma with Gleason score 3 + 3 = 6, in 4/12 biopsies (5 to 10% of their length), 3 in the right lobe (middle part), 1 in the left lobe (apex). MR: hypertrophic prostate, 4 mm, nodular, low-signal intensity on T2, contrast-enhanced, without capsular extension, no other abnormalities	CT and MR	CTV, whole rectum and bladder
**Case two**	**High-risk prostate cancer**. PSA = 35 ng/ml, T3aN0M0 stage. Histology: adenocarcinoma with Gleason score 4 + 4 = 8, in all of the 12 biopsies (60 to 80% of their length). MR: tumor involvement of almost the entire peripheral prostate from the apex to the base, extra capsular extension close to the right base, absence of seminal vesicle invasion, no enlarged lymph node. Bone scan: normal	CT	Pelvic lymph node CTV, small intestine
**Case three**	**Post-operative biochemical failure**. PSA = 0.5 ng/ml, pT3aR1N0 stage. Anatomopathology: adenocarcinoma with Gleason score 3 + 4 = 7, right extra capsular extension, apical positive surgical margin, no lymph node involvement. Pre-operative PSA = 9 ng/ml; time to biochemical failure: 20 months, PSA doubling time 14 months. MR: normal	CT	CTV, whole rectum and bladder

PSA: prostate-specific antigen; CT: computed tomography; MR: magnetic resonance, CTV: clinical target volume

**Table 2:** Definition of index used for the delineation comparison.

Index	Definition	Optimal value
Volume ratio	$\frac{\text{Vn}}{\text{VR}}$	1
Dice similarity coefficient	$2\;\text{x}\frac{{\text{C}}_{\text{n}}\cap {\text{C}}_{\text{R}}}{{\text{C}}_{\text{n}}+{\text{C}}_{\text{R}}}$	1
Overlap	$\frac{{\text{C}}_{\text{n}}\cap {\text{C}}_{\text{R}}}{{\text{C}}_{\text{n}}⋃{\text{C}}_{\text{R}}}$	1
Common Volume	$\frac{{\text{C}}_{\text{n}}\cap {\text{C}}_{\text{R}}}{{\text{C}}_{\text{R}}}$	100%
Additional Volume	$\frac{{\text{C}}_{\text{n}}\;-\;{\text{C}}_{\text{R}}}{{\text{C}}_{\text{n}}}$	0%

VR: volume of the reference contour; Vn: volume of the contour to be compared; CR: reference contour; Cn: contour to be compared.

## Ethics

All of the participating physicians were volunteers. Each one signed a document in which he or she agreed to collaborate on the work (S1 File). This study was financed by several institutions and participating centers (please see the Acknowledgments section) and was administered by the Regional Cancer Network Onco-npdc. According to French laws, this work did not require advice of an ethics committee. Agreement N1034071 was obtained from the "National Commission for Data-collection and Freedom” (‘‘Commission Nationale Informatique et Liberte´”) for the conduct of this work. Anonymized CT and MR images were used for the development of fictitious but realistic clinical cases. D.P. was responsible for anonymizing the data. No participant had access to the patient data prior to anonymization. D.P. was responsible for initially collecting these data. C.V. was responsible for collecting the anonymous results of delineation. None of the authors or participants were involved in the patient’s medical treatment.

## Results

**Fig 1:**
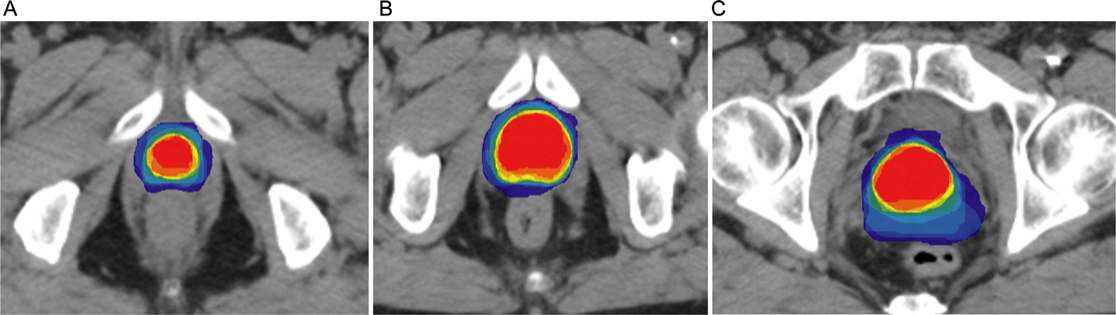
a-c. First comparison of the clinical target volume delineation for case 1: apex (1a), middle prostate (1b) and base (1c).

Fourteen physicians involved in the treatment of urologic cancers at the 11 centers participated. In case 1 (low-risk prostate cancer), the first comparison using method 1 showed acceptable agreement with a DSC value of 0.83 (±0.06). Despite the use of MR images, some differences were observed in the apex and base delineations (Fig 1A, 1B and 1C). The chosen guideline was that by the European Organization for Research and Treatment for Cancer (EORTC) [14]. The indexes were not improved during the second comparison but were considered as correct, with a DSC of 0.83 (±0.08) (Table 3).

**Table 3:** Comparison indexes for the three cases (method 1).

		VR (±SD)	CV (±SD)	AV (±SD)	VO (±SD)	DSC (±SD)
Case 1	Comparison 1	1.16 (±0.26)	89.24 (±7.14)	20.77 (±12.53)	0.71 (±0.08)	0.83 (±0.06)
	Comparison 2	1.11 (±0.19) p = 0.90	87.94 (±7.54) p = 0.67	19.53 (±11.53) p = 0.91	0.72 (±0.10) p = 0.62	0.83 (±0.08) p = 0.62
Case 2	Comparison 1	1.00 (±0.19)	58.34 (±11.20)	41.07 (±10.98)	***0*.*41 (±0*.*09)***	***0*.*58 (±0*.*09)***
	Comparison 2	0.96 (±0.20) p = 0.52	62.11 (±9.91) p = 0.23	33.86 (±9.42) p = 0.07	***0*.*47 (±0*.*07) p = 0*.*009***	***0*.*63(±0*.*07) p = 0*.*0098***
	Comparison 1	***1*.*71 (±0*.*6)***	***83*.*75 (±10*.*77)***	***46*.*58 (±14*.*50)***	0,49 (±0,09)	0,63 (±0,08)
Case 3	Comparison 2	***1*.*34 (±0*.*46) p = 0*.*0034***	***76*.*63 (±16*.*16) p = 0*.*027***	***38*.*08 (±15*.*10) p = 0*.*0024***	0.50 (±0.08) p = 0.96	0.66 (±0.08) p = 0.33

VR: volume ratio; SD: standard deviation; CV: common volume; AV: additional volume; VO: volume overlap; DSC: Dice similarity coefficient. In italics: significant difference.

**Fig 2:**
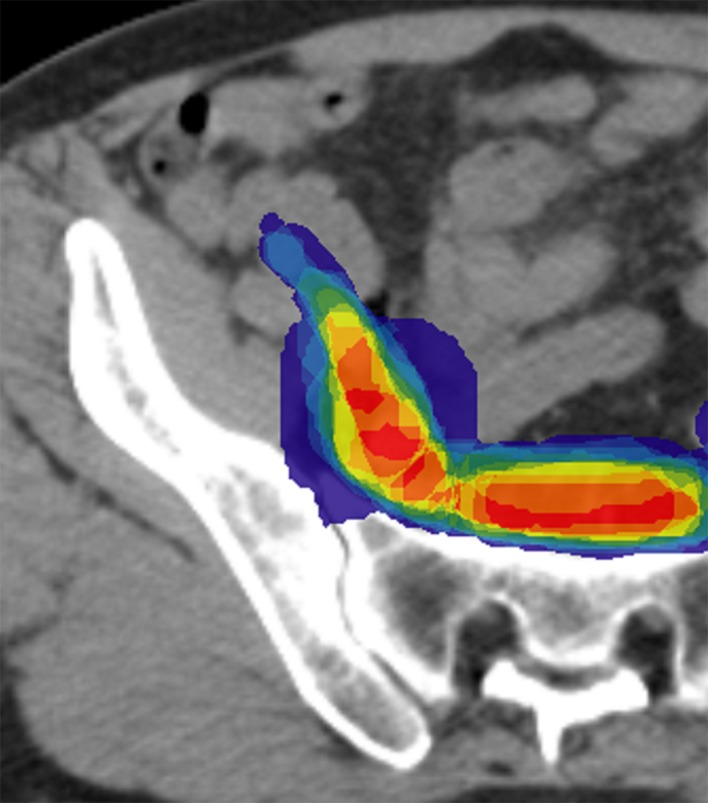
First comparison of the clinical target volume delineation for case 2 (pelvic lymph nodes).

Concerning case 2, the differences in the CTV delineation were mainly located at the inferior and medial borders of the obturator area, the inferior border of pre-sacral and external iliac areas and the superior border of the primitive iliac area (Fig 2). The chosen guidelines were those of the Radiation Therapy Oncology Group (RTOG) [15]. Using method 1 the second comparison showed homogenization of the CTV delineation with a significant improvement in VO (0.41±0.09 vs. 0.47±0.07, p = 0.009) and DSC (0.58±0.09 vs. 0.63±0.07, p = 0.0098) (Table 3). The AV was also improved from 41.07 (±10.98) to 33.86 (±9.42), approaching borderline significance (p = 0.07).

**Fig 3:**
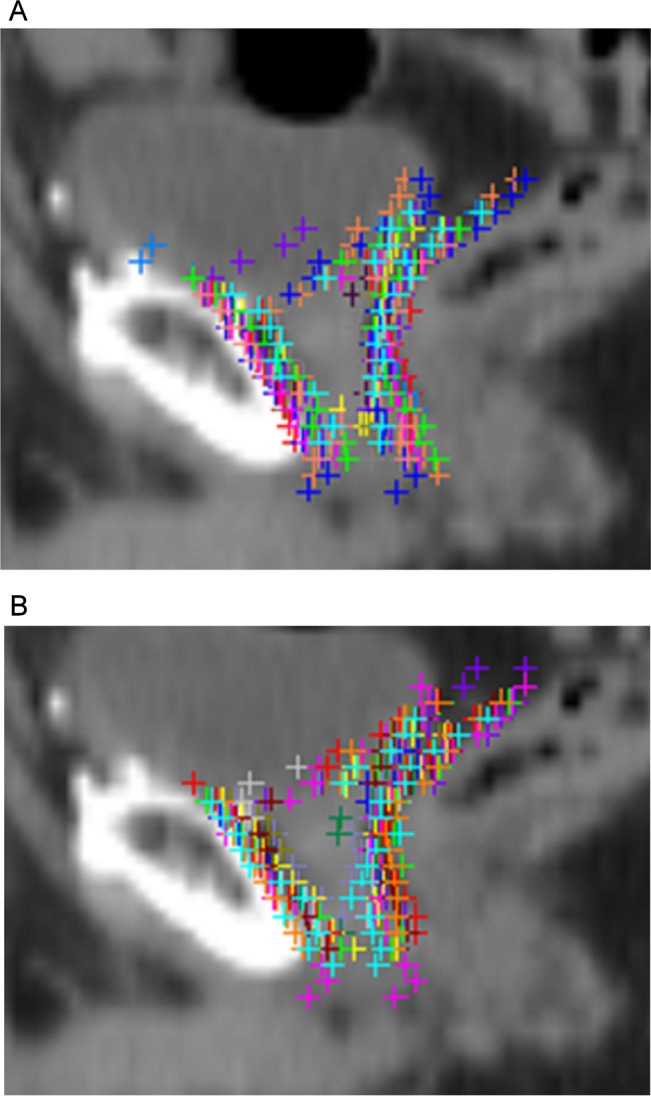
The first (a) and second (b) comparisons of the clinical target volume (CTV) delineation for case 3. Note the homogenization of the delineation of the anterior and superior borders of the CTV.

Concerning case 3, the differences in the CTV delineation were located at the superior and inferior boundaries and at the anterior and superior border of the volume where CTV moves away from the posterior edge of the pubic symphysis (Fig 3). The chosen guidelines were those from the Radiation Therapy Oncology Group (RTOG) [16]. During the second delineation, VR and AV were significantly improved: 1.71 (±0.6) vs. 1.34 (±0.46), p = 0.0034 and 46.58 (±14.50) vs. 38.08 (±15.10), p = 0.0024, respectively using method 1. The CV was probably significantly decreased relative to the large decrease in the volume ratio. DSC was not improved, but it was already superior to 0.6 in the first comparison (Table 3). Analysis of the images showed a standardization of the delineation of the anterior and superior borders of the CTV (Fig 3).

The results were similar using method 2. Concerning case 1, the comparison showed acceptable agreement, with DSC values of 0.84 (±0.07) and 0.85 (±0.09), p = 0.88. None of the other indexes were improved. Concerning case 2, the second comparison showed homogenization of the CTV delineation with a significant improvement in the VO (0.43±0.06 vs. 0.48±0.07, p = 0.05) and DSC (0.6±0.06 vs. 0.65±0.05, p = 0.003). The CV was also improved from 81.4 (±11.7) to 88.6 (±10.26) p = 0.048. Concerning case 3, the AV and VO were significantly improved: 39.3 (±17) vs. 33.9 (±14), p = 0.049 and 0.54 (±0.11) vs. 0.56 (±0.10), p = 0.04, respectively. The CV was not different between the two comparisons (87.13(±10.6) vs. 84.5(±18), p = 0.78). The RV was improved: 1.58 (±0.6) vs. 1.37 (±0.47). However, this difference was not statistically significant (p = 0.068). The DSC was not improved as in method 1, although this index was greater than 0.6 in the first comparison (0.69 (±0.1) vs. 0.71 (±0.08)), p = 0.34.

The VO and DSC were compared between cases 1, 2 and 3 for comparisons 1 and 2, using method 1. These indexes were significantly better in case 1 than in cases 2 and 3 (p<0.05) in comparisons 1 and 2. No significant difference was observed between cases 2 and 3.

## Discussion

The aim of our original work was to prospectively improve the homogeneity of the delineation among all of the senior radiation oncologists in the North of France, regardless of the conditions of practice. To the best of our knowledge, this is the only work of its kind in Europe. In this article, we did not seek to further describe accurately the inter-observer variations, which have already been thoroughly done in the literature, but rather to highlight the qualities of this collaborative work across the Nord-Pas-de-Calais region.

The goal of the present study was to evaluate the homogenization of delineation among physicians. There is no standard method in literature for this work; thus, a reference is necessary to calculate the indexes. We hypothesized that the random selection of the same physician would not significantly influence the results. Concerning the “reference” contours from one physician, the differences were slight between the first and second delineation (data not shown). As a limitation of the present study, we could not assert whether this hypothesis was completely right. To overcome this limitation, we compared each delineation with a common contour comprising the delineations of most of the physicians. This method facilitated the evaluation of the harmonization of delineation and avoided the selection of a random or reference contour. The results were similar whatever the method used, with the improvement of some indexes for cases 2 and 3.

It is important to note that the volumetric indexes used in our study to compare the CTV delineation are more sensitive than metric ones. For example, the volume overlap (VO) of two volumes overlapping at 85% is 0.74. The VO of two cubes composed of 10×10×10 voxels after the shifting of one voxel along the diagonal of the cube is 0.57 (729/1271), whereas the mean distance between the two cubes is around one voxel only [11]. There is no standard value beyond the inter-observer variation that is considered low. It is commonly accepted that a value greater than 0.6 is correct; a value greater than 0.8 is considered good and close to the intra-observer variability. In the present study, the DSC values were superior to 0.6 in cases 1 and 3 in the first comparison and after the second comparison in case 2 using method 1. The indexes were not improved in case 1, for which the inter-observer agreement was considered good after the first comparison whatever the method used. Some indexes were improved during the second comparison (method 1: VO and DSC in case 2, VR and AV in case 3; method 2: CV, VO and DSC in case 2, VO and AV in case 3).

The inter-observer delineation variation was significantly larger in cases 2 and 3 than in case 1 for the two comparisons. Indeed, the complexity of these cases was more important, with a delineation based on the pelvic vascular anatomy for case 2 and the lack of macroscopic target for case 3.

Inter-observer delineation variation and its influence on dosimetry have been shown for many years in almost every tumor location [5–7,17–21]. Multimodality fusion can improve homogeneity [22–24]. Some studies have shown an improvement in the delineation homogeneity between radiation oncology residents after educational intervention [25,26]. Short-term improvement in head and neck delineation was shown in 11 residents after a teaching intervention; in this study, the evaluation was subjective as contours were scored in a blinded fashion by the investigators [26]. Wide heterogeneity can be observed among the senior radiation oncologists. In the study by Lawton et al., significant disagreement existed in the definition of the CTV for pelvic nodal radiation therapy among genito-urinary radiation oncology experts [7], leading to the development of a consensus [15]. Nevertheless, in some situations, guidelines may vary. Malone et al. compared four consensus guidelines concerning the CTV delineation for post-operative radiotherapy after prostatectomy in 20 patients. The mean volumes (±SD) were 60 (±17) cc and 102 (±24) cc for the smaller and larger ones, respectively, bringing about large differences in the doses delivered to OARs [27].

From this statement, scientific societies have implemented delineation courses worldwide; closer to our region, we can mention the online European and French tools as well as the training delivered during their annual conferences [28–31]. The originality of our additional work lies in the prospective exchange and collaboration of all physicians across our region in a formal setting. This work is ongoing with head and neck and breast delineation and a comparison of prostate cancer intensity-modulated radiotherapy optimization based on common volumes. We wish to extend our work to our neighboring region, Picardy, with which a merger is planned.

## Conclusion

This prospective study showed that a collaborative discussion concerning clinical cases and the selection of shared guidelines within an established framework improved the homogeneity of the CTV delineation among the senior radiation oncologists in the Nord-Pas-de-Calais region.

## Supporting Information

S1 File Physician agreement. (PDF)
