# Supplementary material for: Harmonization of the Volume of Interest Delineation among All Eleven Radiotherapy Centers in the North of France
Source: PLoS One. 2016 Mar 17;11(3):e0150917. doi: 10.1371/journal.pone.0150917 (PMC4795685; doi:10.1371/journal.pone.0150917)
Supplement: S1 File — (PDF) [file pone.0150917.s001.pdf]

AVENANT A LA CONVENTION DE MISE A DISPOSITION ET D'AVANCE DE FRAIS

Préambule

L'association ONCO NORD PAS DE CALAIS, gestionnaire du Réseau Régional de Cancérologie du Nord Pas de Calais, est porteur financier du projet « d'harmonisation des pratiques en radiothérapie » initié par l'Association CRONOR. Ce projet prévoit la mise à disposition par l'association ONCONPDC d'une station de travail ARTIVIEW P2E dans chaque centre de radiothérapie de la région Nord Pas de Calais. Le plan de financement prévoit d'une part, des prestations récurrentes de support d'assistance et de maintenance à la charge des centres de radiothérapie et d'autre part, l'échange de données médicales entre praticiens via une plateforme de communication sécurisée et cryptée garantissant la confidentialité du transfert des données, conformément à la règlementation en vigueur.

En conséquence,

Entre

L'association ONCO Nord Pas-de-Calais, sise, 180 rue Eugène Avinée – Parc Eurasanté Ouest – 59120 LOOS, représentée par son Président, Monsieur le Pr Jacques Bonneterre, dûment habilité,

D'une part

Et

Le Centre de radiothérapie . . . . dont le siège est situé au . . . . , représenté par le Dr . . . .,

D'autre part

Il est convenu et arrêté ce qui suit :

Article 1 – Objet de la convention

La présente convention fixe les modalités de versement de la participation financière du Centre . . . . à la maintenance de la station de travail ARTIVIEW P2E et à la gestion de la plateforme de communication sécurisée et cryptée (devis en annexe).

Article 2 – Engagements - Responsabilités

Le Réseau Régional s'engage à fournir une station de travail ARTIVIEW P2E, un accès à la plateforme d'échange sécurisé, ainsi que la mise à disposition d'un chef de projet.

Le centre de radiothérapie est responsable de la station de travail qui lui est confiée et prend toute mesure de protection et d'assurance nécessaire.

Article 3 – Durée de la convention

La présente convention est conclue pour une durée indéterminée, liée à la durée de l'action menée par l'association. Elle prend effet à la date de signature soit le . . . . et prendra fin à l'arrêt du versement par l'ARS de la subvention correspondant à la rémunération du technicien.

Article 4 - Modalités de versement des crédits

La participation annuelle de chaque centre de radiothérapie est fixée, en 2014, à 8435,79€ par station de travail mise à disposition, incluant le droit de licence valable pour une année, l'assistance sur site, l'assistance téléphonique, la maintenance logicielle et matérielle ainsi que la gestion et l'hébergement des données de la plateforme sécurisée et cryptée.

Ce versement est subordonné à la signature de la présente convention, et sera effectué auprès du comptable assignataire de l'association ONCO Nord Pas-de-Calais par virement bancaire.

Article 5 – Litiges

Les Tribunaux de Lille sont compétents pour tout litige relatif à l'exécution de la présente convention.

Fait à Loos, le . . . .
En deux exemplaires originaux,

Pour l'Association
ONCO Nord Pas-de-Calais
Pr Jacques Bonneterre,
Président

Pour le
Centre . . . .
Dr . . . .
